# Effect of exercise on Fibroblast Growth Factor 21 levels in healthy males and females

**DOI:** 10.1371/journal.pone.0321738

**Published:** 2025-05-29

**Authors:** Matthew Peterson, Kathleen A. Richardson, LesLee Funderburk

**Affiliations:** 1 Department of Health, Human Performance, and Recreation, Baylor University, Waco, Texas, United States of America; 2 Human Sciences and Design, Baylor University, Waco, Texas, United States of America; University of Navarra School of Medicine and Center for Applied Medical Research (CIMA), SPAIN

## Abstract

Fibroblast growth factor 21 (FGF21) is a metabolic regulator that may increase in circulation following exercise. This study was undertaken to determine if changes to FGF21 post-exercise are dependent on biological sex and exercise modality. Secondary analyses were conducted to determine if the post-exercise rise in FGF21 was associated with upstream signaling factors (glucagon, epinephrine, glucose) and excess post-exercise oxygen consumption (EPOC) response. Following a randomized crossover design, male and female participants completed two, thirty minute exercise protocols – during the steady state (SS) protocol, participants cycled at 70% of VO_2peak_ and during the sprint interval exercise (IE) protocol participants completed six, thirty second “all out” sprints against 7.5% of body weight with four and half minutes of active recovery between sprints. Blood samples were taken at baseline, immediately post-exercise (IPE), and 1-hour post-exercise. Oxygen consumption was monitored continuously throughout the trials. In males, FGF21 levels were increased at 1-hour post-exercise with no difference between conditions; in females, FGF21 levels were unchanged as a result of exercise. Comparison of the post-exercise FGF21 incremental area under the curve revealed that in the SS condition males had a greater FGF21 response than females and that within males the SS condition had a greater response than the IE condition. Exercise significantly increased FGF21 levels in males, but not in females. In males, FGF21 levels were greater in the SS condition than in the IE condition.

## 1. Introduction

Fibroblast growth factor 21 (FGF21) is a circulating signaling factor that has broad actions in metabolism. Originally identified for its ability to promote glucose uptake [1], a derivative of FGF21 has also been shown to promote weight loss and improve dyslipidemia when given as a pharmacological agent [2]. These observations have created a significant amount of research interest in FGF21 over the past decade.

Many metabolic characteristics display a bimodal distribution in humans based on biological sex. FGF21 may play a role in these sex differences. In mice, FGF21 might disrupt female fertility [3]. In humans sex based differences in FGF21 have been observed in response to hyperinsulinemia [4] and fructose overfeeding [5]. Resting FGF21 concentrations are similar between females and males [6]; however, exercise presents a metabolic demand that may expose sex differences not present at rest.

One major research finding is that serum FGF21 levels are increased following an acute bout of aerobic exercise [7–14] and that this rise is primarily the result of hepatic excretion [9]. Time course studies have shown that this increase peaks at one hour post-exercise [8,14]. A variety of exercise intensities have been demonstrated to bring about this post-exercise increase in FGF21, but the changes appear to be more robust with a greater exercise intensity [7,11,12]. While aerobic exercise has consistently been shown to increase serum FGF21 levels post-exercise, the effect of other types of exercise such as sprint interval exercise has not been fully evaluated. Sprint interval exercise consists of short bouts of anaerobic sprints followed by low-intensity recovery periods and can have similar metabolic benefits to aerobic exercise [15,16].

The regulation of FGF21 is a complex process, with many paradoxical signaling pathways [17]. Many of the signaling factors that stimulate FGF21 production are also upregulated during exercise. Prior work has shown that a spike in blood glucagon levels precedes a rise in FGF21 levels following exercise [8]. The effect of other upstream signaling pathways such as epinephrine and glucose is not as established. Changes in epinephrine and glucose flux during exercise have been demonstrated to exhibit sex based differences [18,19] which might contribute to sex based differences in exercise induced FGF21 concentrations.

The purpose of this study was to investigate the effect of aerobic and sprint interval exercise on serum levels of FGF21 in humans. We hypothesized that the post-exercise change in FGF21 concentrations would exhibit sex-based differences and be related to upstream signaling molecules and post-exercise metabolism.

## 2. Materials and methods

### 2.1. Study population

Male and female participants were recruited for this study from April 1, 2019 to December 1, 2019. Inclusion criteria included: age between 18 and 45 years old, regular exercisers (at least thirty minutes of exercise three days a week), a BMI between 18.5 and 24.9, and free from any cardiovascular or metabolic diseases. Female participants also had to be eumenorrheic and not using hormonal contraceptives.

All procedures were approved by the Baylor University Institutional Review Board (Reference # 1406758). Before starting the study, all participants were informed of the study procedures and provided written informed consent. All procedures were conducted in compliance with the declaration of Helsinki.

### 2.2. Experimental design

We utilized a randomized cross-over design. Participants made three separate visits to the laboratory. During visit 1 we measured body composition using dual energy x-ray absorptiometry (DEXA) (Hologic Discovery Series W; Waltham, MA) and administered a peak oxygen consumption (VO_2peak_) test. The VO_2peak_ test was conducted using a mechanically braked bike (Lode; Groningen, Netherlands) and respiratory gas analysis system (TrueOne 2400, Parvo Medics; Salt Lake City, UT). The VO_2peak_ test started at a resistance of 50W and the resistance was increased by 50W every two minutes, participants maintained a cadence of 60rpm for the entirety of the test. The test was terminated when the participant experienced volitional fatigue or the pedaling cadence fell below 60rpm for thirty consecutive seconds (whichever came first). VO_2peak_ was confirmed by a respiratory exchange ratio (RER) above 1.1 and plateau in VO_2_. The highest thirty second average oxygen consumption was used to determine subsequent workloads.

Participants completed visits 2 and 3 following a randomized crossover design. Randomization was determined based on the order of study enrollment. The day prior to visits 2 and 3, participants were asked to keep a similar diet, exercise, and sleep pattern that was free of ethanol and vigorous activity; participants were also asked to arrive to visit 2 and 3 after a 12-hour overnight fast. These procedures were confirmed by having participants complete a 24-hour dietary recall (ASA24) prior to each visit and by wearing an activity and sleep monitor (SenseWear by Bodymedia; Pittsburgh, PA). Respiratory gasses and heart rate (HR) (H7, Polar; Kempele, Finland) were continuously monitored during visits 2 and 3, and the two visits were identical except for the exercise session. Both visits started with the participant resting in a supine position for fifteen minutes. A blood sample was obtained during the first five minutes of this resting period and the final five minutes of respiratory gas analysis were averaged to obtain baseline values. Following the resting measures, participants cycled for five-minutes at 40% of their VO_2peak_ to warm-up. The exercise bout began immediately after the warm-up: Exercise sessions were matched for time at 30 minutes to meet the current exercise recommendations [20]. During the steady state exercise (SS) condition, participants cycled at 70% of their VO_2peak_ for 30 minutes. For the sprint interval exercise (IE) condition, participants completed six bouts of 30 second “all out” sprints against a resistance equal to 7.5% of their body weight; a 4.5-minute active recovery period of unloaded cycling followed each sprint. Immediately following the exercise session, participants returned to a supine position and an immediately post-exercise (IPE) blood sample was obtained. Participants continued to rest in a supine position for 1-hour post-exercise. At 1-hour post-exercise a third and final blood sample was obtained.

Visits 2 and 3 were separated by a minimum of 24 hours. Female participants completed visits 2 and 3 during the mid-follicular stage of their menstrual cycle (days 3–10).

### 2.3. Blood sampling

Blood samples were procured from the most prominent vein of the antecubital space at baseline, IPE, and 1-hour post-exercise for each experimental trial. Both serum and plasma (KEDTA) were collected. Blood was allowed to sit at room temperature for thirty minutes and then centrifuged at 3,000 rpm for fifteen minutes. Serum and plasma were separated and stored at -80°C until analysis.

Serum FGF21 (DF2100, R&D Systems), serum glucagon (ELH-Glucagon, RayBio), and plasma epinephrine (NBP2–62867, Novus Biologicals) were measured using commercially available ELISAs. Serum glucose measurements were completed and reported by Clinical Pathology Laboratories (Waco, TX) using the Roche COBAS automated methodology.

### 2.4. Statistics

Sample size was determined *a priori* by evaluating typical changes to blood FGF21 concentrations following exercise. Sample size estimates indicated that 10 females and 10 males would be sufficient to examine sex differences. Accounting for dropouts and the potential for FGF21 levels to be undetectable in some individuals, recruitment continued until fifteen females and fifteen males completed all aspects of the study. Sample size calculations were done using G*Power (Düsseldorf, Germany).

Blood markers that were below the limit of detection were treated as missing variables using pairwise deletion. Blood variables were checked for normality using the Shapiro-Wilk test and those that violated normality were natural log transformed prior to analysis. We used a 2 by 3 (condition by time) mixed model ANOVA with repeated measures to investigate changes to blood variables across time and condition. Significant ANOVA findings were followed up using post-hoc tests with a Bonferroni adjustment for multiple comparisons. Dependent *t-*tests and independent *t*-tests were used to compare baseline values between conditions and between females and males, respectively. Pearson correlations were used to investigate the relationships between FGF21 and other circulating biomarkers. FGF21 incremental area under the curve (iAUC) and total area under the curve (tAUC) were calculated using the trapezoidal rule. The AUC values contained negative numbers and could not be log transformed; therefore, they were analyzed using appropriate non-parametric tests. All analyses were conducted using SPSS version 25 (SPSS, Chicago, Illinois, USA). An alpha level of *p* < 0.05 was adopted throughout. Raw data are available at https://doi.org/10.6084/m9.figshare.28425968.v1.

## 3. Results

### 3.1. Participants’ characteristics

**Fig 1:**
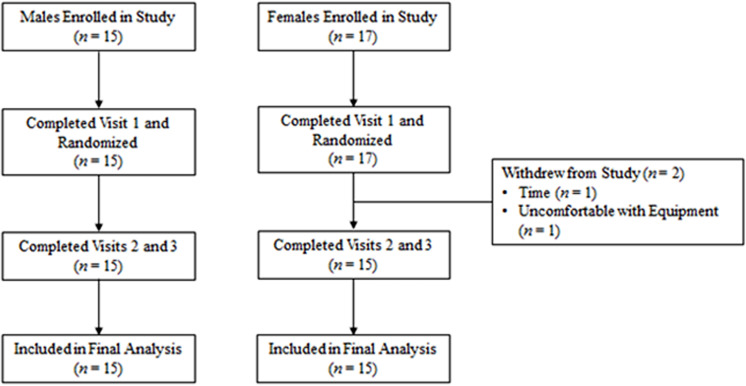
CONSORT flow diagram.

Seventeen female participants and fifteen male participants provided informed consent and completed visit 1. One female participant withdrew due to time constraints and another due to feeling uncomfortable with the respiratory gas analysis mask, all others completed this study (Fig 1) The participants were compliant with study procedures as dietary recalls, activity, and sleep patterns were all similar between experimental trials (*p* > 0.05). Demographic characteristics are presented in Table 1.

**Table 1:** Demographic characteristics.

Characteristic	Female (*n* = 15)	Male (*n* = 15)
Age	23.5 ± 4.3	26.9 ± 6.5
VO_2peak_	30.8 ± 5.5	45.1 ± 5.3
Height	164 ± 6.2	178 ± 8.3
Weight	58.5 ± 8.7	74.4 ± 6.9
BMI	21.4 ± 1.9	23.4 ± 1.3
%BF	25.7 ± 3.0	15.4 ± 4.8

Data are presented as mean ± SD. Age (years); VO2peak, peak oxygen consumption (mL/kg/min); BMI, body mass index (kg/m2); %BF, percent body fat.

### 3.2. Exercise sessions

During the SS session, the average intensity for females was 67 ± 0.01% of VO_2peak_, for males the average intensity was 68 ± 0.02% of VO_2peak_, these differences were not statistically significant (*t *= -0.48, *p* = 0.64). Due to feelings of nausea, two males only completed five of the six sprints during the IE condition. Average values for the exercise variables are presented in Table 2.

**Table 2:** Exercise variables.

Variable	SS		IE	
	Females (*n* = 15)	Males (*n* = 15)	Females (*n* = 15)	Males (*n* = 15)
VO_2_R	20.6 ± 4.1 ^*^^,^^#^	30.5 ± 4.6 ^*^^,^^#^	14.1 ± 3.0 ^*^^,^^#^	18.1 ± 3.2 ^*^^,^^#^
VO_2_A	1.2 ± 0.3 ^*^^,^^#^	2.3 ± 0.4 ^*^^,^^#^	0.8 ± 0.2 ^*^^,^^#^	1.4 ± 0.3 ^*^^,^^#^
VE	30.8 ± 8.3 ^*^	54.2 ± 10.4 ^*^^,^^#^	27.8 ± 7.0 ^*^	47.6 ± 13.2 ^*^^,^^#^
RER	0.96 ± 0.03 ^#^	0.96 ± 0.03 ^#^	1.10 ± 0.05 ^*^^,^^#^	1.17 ± 0.05 ^*^^,^^#^
HR	149.2 ± 18.4 ^#^	157.0 ± 13.6 ^#^	132.9 ± 22.7 ^#^	138.4 ± 21.4^#^
EE	179.2 ± 43.5 ^*^^,^^#^	338.9 ± 57.8 ^*^^,^^#^	124.3 ± 26.5 ^*^^,^^#^	204.9 ± 49.7 ^*^^,^^#^
Distance cycled	9.2 ± 2.2 ^*^^,^^#^	16.8 ± 3.2 ^*^^,^^#^	3.5 ± 0.6^*^^,^^#^	5.6 ± 1.0 ^*^^,^^#^

Data are presented as mean ± SD,

* = statistically significant difference between males and females,

# = statistically significant difference between SS and IE. VO2R, relative oxygen consumption (mL/kg/min); VO2A, absolute oxygen consumption; (L/min), VE, minute ventilation (L/min); RER, respiratory exchange ratio (VCO2/VO2); HR, heart rate (BPM); EE, Energy Expenditure (kcal); Distance cycled (km).

### 3.3. FGF21

**Fig 2:**
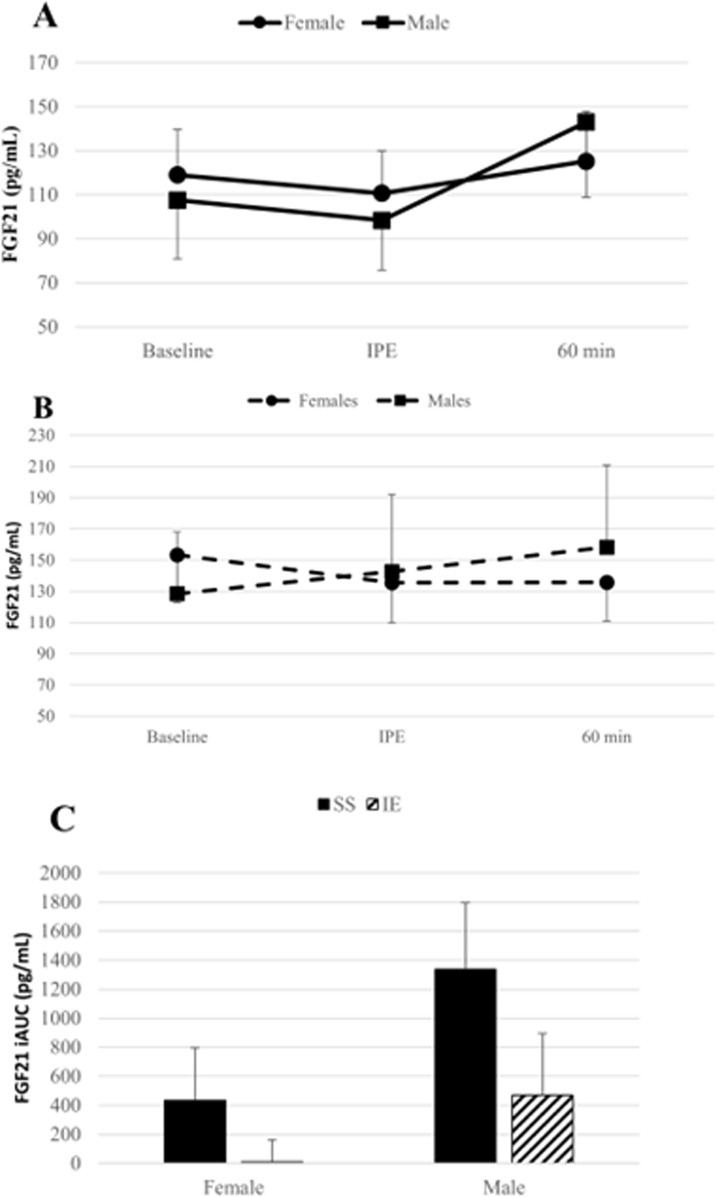
FGF21 response to exercise. Data graphed as mean±SEM **A**. FGF21 response to SS in females (*n* = 13) and males (*n* = 12). **B**. FGF21 response to IE in females (*n* = 13) and males (*n* = 11). **C**. FGF21 post-exercise iAUC in males and females following SS and IE. * = significantly elevated relative to baseline, a = significantly different between males and females, # = significantly different between SS and IE.

Fasting concentrations of FGF21 were not different between females and males at baseline (*p* < 0.05). For females, fasting concentrations of FGF21 were 119 ± 21 pg/mL and 153 ± 31 pg/mL before SS and IE exercise, respectively and were not significantly different between conditions (*p* > 0.05). Immediately post-exercise, FGF21 concentrations were decreased relative to baseline. In the SS condition, FGF21 levels rose above baseline to 125 ± 23 pg/mL 1-hour post exercise, while in the IE condition, FGF21 levels remained depressed relative to baseline. ANOVA results indicated that there were no statistically significant main effects for time (*F*_*(2,24)*_ = 0.59, *p* = 0.48, η_p_^2^ = 0.05 [90% CI = 0.00–0.17]) or condition (*F*_*(1,12)*_ = 0.40, *p* = 0.54, η_p_^2^ = 0.03 [90% CI = 0.00–0.26]) (Fig 2A and 2B).

For males, fasting concentrations of FGF21 were 108 ± 27 pg/mL and 128 ± 39 pg/mL prior to SS and IE exercise, respectively, and were not different between conditions (*p* > 0.05). Serum FGF21 concentrations decreased slightly immediately after SS exercise and were elevated immediately post-exercise during the IE condition. An hour after exercise, FGF21 levels were elevated in both conditions, rising to 143 ± 34 pg/mL after SS and 158 ± 52 after IE. There was a statistically significant effect for time (*F*_*(2,20)*_ = 10.64, *p* < 0.01, η_p_^2^ = 0.52 [90% CI = 0.20–0.65]). Neither the main effect for condition (*F*_*(1,10)*_ = 0.12, *p* = 0.74, η_p_^2^ = 0.01 [90% CI = 0.00–0.22]) nor the condition by time interaction effect (*F*_*(2,20)*_ = 1.45, *p* = 0.26, η_p_^2^ = 0.13 [90% CI = 0.00–0.30]) were statistically significant. Post-hoc tests revealed that 1-hour post-exercise was significantly elevated relative to the baseline and immediately post-exercise time points (*p* < 0.05) (Fig 2A and 2B).

The post-exercise iAUCs were compared between sexes and between conditions. During the SS condition the post-exercise iAUC was significantly greater in males than in females (*U = *46.0, *p* = 0.04) (Fig 2C). While males had a greater post-exercise iAUC than females during the IE condition, this difference was not statistically significant (*U* = 53.0, *p* = 0.14) (Fig 2C). For females, there was not a significant difference between conditions (*Z* = -1.22, *p* = 0.22). For males, the SS condition produced a significantly greater post-exercise FGF21 than the IE condition (*Z *= -2.3, *p* = 0.02) (Fig 2C).

### 3.4. Glucagon, epinephrine, glucose

Glucagon, epinephrine, and glucose are all mechanistically linked to FGF21 production. Baseline values for glucagon and epinephrine were not significantly different between males and females (*p* > 0.05). Fasting blood glucose was significantly higher in males before the SS condition (*p *< 0.05), but not significantly different between sexes before the IE condition (*p* > 0.05).

**Fig 3:**
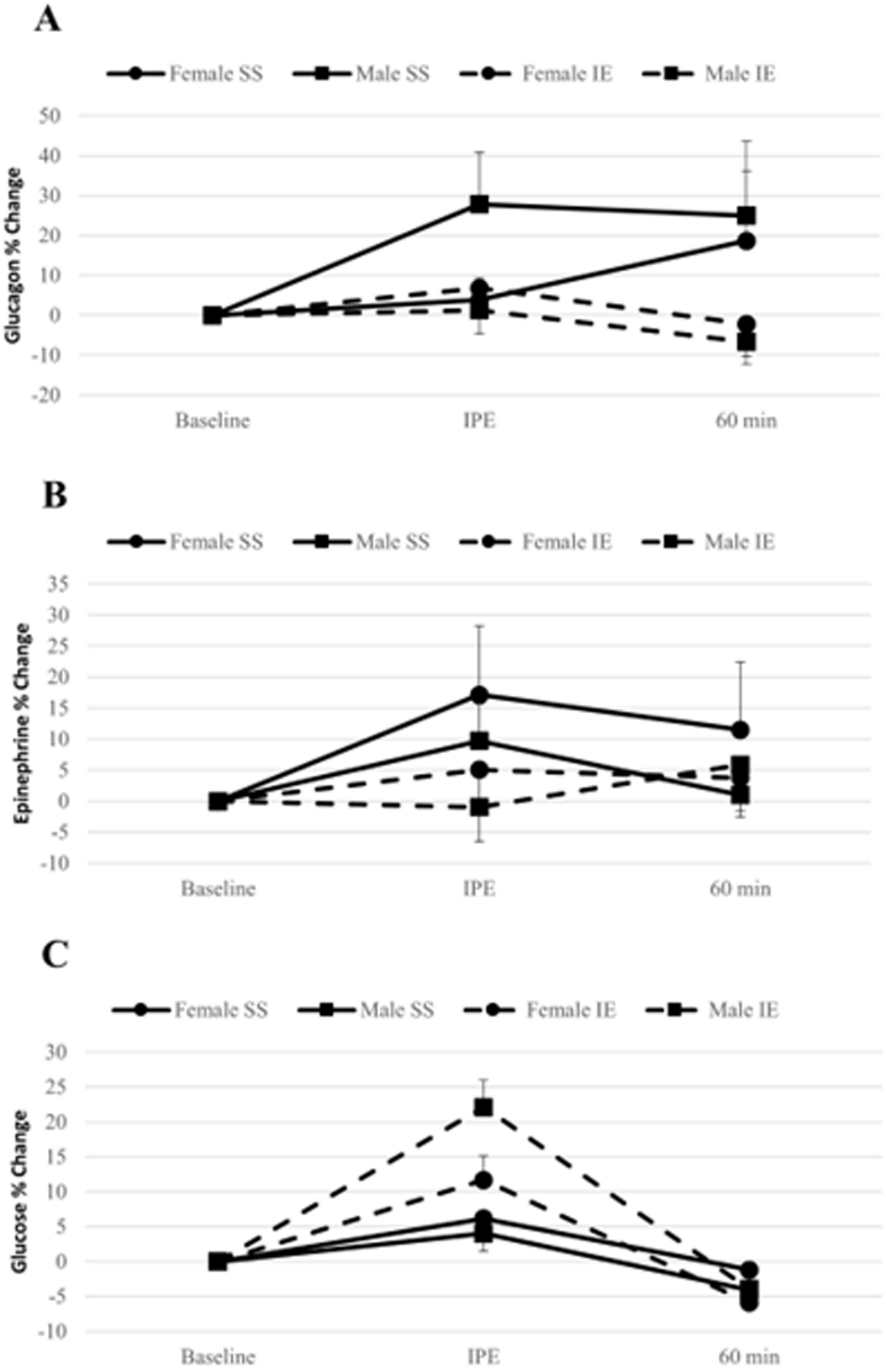
Signaling factors response to exercise. Data graphed as mean±SEM **A**. Glucagon percent change with exercise in females (*n* = 15) and males (*n* = 14). **B**. Epinephrine percent change with exercise in females (*n* = 14) and males (*n* = 15). **C**. Glucose percent change with exercise in females (*n* = 15) and males (*n* = 15). * = significantly elevated relative to baseline, # = significantly depressed relative to baseline.

Glucagon levels changed as a result of time, increasing during the SS trial and decreasing during the IE trial in both males and females; however, these changes were not statistically significant [Females (*F*_*(2,28)*_ = 0.46, *p* = 0.64, η_p_^2^ = 0.03 [90% CI = 0.00–0.14]); Males (*F*_*(2,26)*_ = 0.95, *p* = 0.37, η_p_^2^ = 0.07 [90% CI = 0.00–0.21])]. Additionally, changes to glucagon levels were not significantly different between conditions [Females (*F*_*(1,14)*_ = 0.67, *p* = 0.8, η_p_^2^ = 0.00 [90% CI = 0.00–0.15]); Males (*F*_*(1,13)*_ = 1.66, *p* = 0.22, η_p_^2^ = 0.11 [90% CI = 0.00–0.37])] (Fig 3A).

In females, epinephrine was elevated immediately post-exercise, but this change was not statistically significant (*F*_*(2,26)*_ = 1.70, *p* = 0.22, η_p_^2^ = 0.12 [90% CI = 0.00–0.28]), nor were changes to epinephrine levels influenced by exercise condition (*F*_*(1,13)*_ = 0.00, *p* = 0.97, η_p_^2^ = 0.00 [90% CI = 0.00–0.01]). Similar results were found in males, where epinephrine levels were not significantly altered across time (*F*_*(2,28)*_ = 0.30, *p* = 0.74, η_p_^2^ = 0.02 [90% CI = 0.00–0.11]) or condition (*F*_*(1,14)*_ = 0.12, *p* = 0.73, η_p_^2^ = 0.01 [90% CI = 0.00–0.18]) (Fig 3B).

For both sexes, blood glucose levels were elevated immediately post-exercise and returned to baseline levels or below by 1-hour post exercise in both conditions. In females, there was a significant main effect for time (*F*_*(2,28)*_ = 16.90, *p* < 0.001, η_p_^2^ = 0.55 [90% CI = 0.29–0.66]). There was not a significant main effect for condition (*F*_*(1,14)*_ = 3.52, *p* = 0.08, η_p_^2^ = 0.20 [90% CI = 0.00–0.45]), but there was a significant time by condition interaction effect (*F*_*(2,28)*_ = 4.46, *p* = 0.04, η_p_^2^ = 0.24 [90% CI = 0.02–0.41]). Post-hoc tests showed that the immediately post-exercise levels were elevated relative to baseline (*F*_*(1,14)*_ = 13.83, *p* < 0.01, η_p_^2^ = 0.50 [90% CI = 0.15–0.67]), that 1-hour post exercise levels were depressed relative to baseline (*F*_*(1,14)*_ = 11.14, *p* < 0.01, η_p_^2^ = 0.44 [90% CI = 0.10–0.63]), and that the change from baseline to 1-hour post-exercise was different between conditions (*F*_*(1,14)*_ = 10.68, *p* < 0.01, η_p_^2^ = 0.43 [90% CI = 0.09–0.62]). In males, similar results were witnessed with a significant main effect for time (*F*_*(2,28)*_ = 28.50, *p* < 0.001, η_p_^2^ = 0.67 [90% CI = 0.45–0.76]) and significant time by condition interaction (*F*_*(2,28)*_ = 14.19, *p* < 0.001, η_p_^2^ = 0.50 [90% CI = 0.24–0.63]), but no main effect for condition (*F*_*(1,14)*_ = 3.74, *p* = 0.07, η_p_^2^ = 0.21 [90% CI = 0.00–0.45]). Post-hoc analysis indicated that the immediately post-exercise blood glucose levels were greater than baseline values (*F*_*(1,14)*_ = 28.90, *p* < 0.001, η_p_^2^ = 0.67 [90% CI = 0.36–0.79]) and that the change from baseline to immediately post-exercise was significantly different between conditions (*F*_*(1,14)*_ = 15.39, *p* < 0.01, η_p_^2^ = 0.52 [90% CI = 0.17–0.69]). In men blood glucose levels 1-hour after exercise were not significantly different from pre-exercise values (*F*_*(1,14)*_ = 4.28, *p* = 0.06, η_p_^2^ = 0.23 [90% CI = 0.00–0.47]) (Fig 3C).

### 3.5. Relationship between FGF21 and other blood markers

To examine the relationship between exercise induced changes to FGF21 signaling factors and FGF21, we conducted correlations between the IPE concentration of glucagon, epinephrine, and glucose and the post-exercise FGF21 iAUC. Glucose was significantly correlated with a strong effect size to the FGF21 iAUC in males during the IE protocol (*r* = 0.71, *p* < 0.01, [95% CI = 0.19–0.92]). No other statistically significant correlations were observed (Table 3).

**Table 3:** Correlations between signaling factors and FGF21.

Condition	Correlate	Females		Males		Whole Group	
		*r*	*p*	*r*	*p*	*r*	*p*
SS	Glucagon	-0.08	0.40	-0.19	0.28	-0.07	0.037
	Epinephrine	0.06	0.43	0.32	0.16	0.15	0.24
	Glucose	-0.19	0.27	0.31	0.16	0.12	0.28
IE	Glucagon	0.19	0.26	-0.43	0.11	-0.17	0.23
	Epinephrine	0.05	0.44	-0.13	0.35	-0.09	0.34
	Glucose	0.36	0.11	0.71	<0.01*	0.63	<0.01^*^

The IPE signaling factor concentration was correlated to the post-exercise FGF21 iAUC.

* = statistically significant finding.

### 3.6. EPOC

Following the exercise session, we continued to monitor oxygen consumption for 1-hour post-exercise. After the SS condition, both the caloric expenditure attributed to the EPOC response (EPOC magnitude) (*t *= -6.45, *p* < 0.001) and RER (*t *= 2.49, *p* = 0.02) were significan*t*ly different between females and males. Similar sex-based differences were observed after the IE condition – EPOC magnitude (*t *= -4.77, *p* < 0.001) and RER (*t *= 3.30, *p* < 0.01). Analyzing females and males separately indica*t*ed that EPOC magnitude was not different between condition (Females: *t *= -0.34, *p* = 0.74; Males: *t *= -0.28, *p* = 0.78), but RER was significan*t*ly grea*t*er following the SS condition (Females: *t *= 6.45, *p* < 0.001; Males: *t *= 4.77, *p* < 0.001) (Table 4).

**Table 4:** EPOC variables.

Variable	SS		IE	
	Females	Males	Females	Males
EPOC magnitude	8.88 ± 1.74 ^*^	24.06 ± 1.60 ^*^	9.59 ± 1.99 ^*^	24.79 ± 2.49 ^*^
RER	0.86 ± 0.01 ^*^^,^^#^	0.81 ± 0.01 ^*^^,^^#^	0.79 ± 0.01 ^*^^,^^#^	0.75 ± 0.01 ^*^^,^^#^

Data are presented as mean ± SEM,

* = statistically significant difference between males and females,

# = statistically significant difference between SS and IE conditions. EPOC magnitude, excess post-exercise oxygen consumption (kcal); RER, respiratory exchange ratio (VCO_2_/VO_2_).

### 3.7. Relationship between EPOC and FGF21

We next sought to determine if the post-exercise FGF21 response was related to EPOC magnitude or RER. We compared the post-exercise FGF21 iAUC with the total EPOC magnitude (kcal) and absolute VO_2_ and RER during the final five minutes of the post-exercise recovery period. No statistically significant correlations were found between the FGF21 iAUC and EPOC variables (Table 5).

**Table 5:** Correlations between EPOC variables and the post-exercise FGF21 iAUC.

Condition	Correlate	Females		Males		Whole Group	
		*r*	*p*	*r*	*p*	*r*	*p*
SS	VO_2_A	-0.13	0.34	0.23	0.24	0.29	0.08
	RER	-0.17	0.29	-0.04	0.45	-0.22	0.15
	EPOC magnitude	0.28	0.17	-0.02	0.47	0.31	0.06
IE	VO_2_A	0.33	0.14	0.088	0.40	0.27	0.11
	RER	-0.03	0.46	0.25	0.23	-0.04	0.42
	EPOC magnitude	0.30	0.16	0.08	0.41	0.26	0.11

FGF21 iAUC, FGF21 incremental area under the curve, VO2A, absolute oxygen consumption (L/min) recorded during the final five minutes of the exercise recovery period; RER, respiratory exchange ratio (VCO_2_/VO_2_) recorded during the final five minutes of the exercise recovery period; EPOC magnitude, energy expenditure during the post-exercise recovery period due to Excess Post-exercise Oxygen Consumption.

## 4. Discussion

The present study explored the effect of an acute bout of sprint interval or continuous exercise on circulating levels of FGF21 and related biomarkers as well as EPOC in healthy individuals. Our novel findings are that exercise elevates FGF21 post-exercise to a greater degree in males than it does in females, that continuous exercise produces greater levels of circulating FGF21 than sprint interval exercise, and that FGF21 is not related to the EPOC response.

Sex differences in the exercise response to FGF21 are a notable finding and might help explain some of the metabolic differences observed between males and females. Steady state endurance exercise has consistently been shown to acutely increase FGF21 in healthy men [7–14]. Similar to these findings, we saw a statistically significant increase in FGF21 following the SS exercise protocol in our cohort of males, but in females this response was not significantly elevated. Furthermore, we even saw a decrease in FGF21 levels (relative to baseline) in females following the IE condition. Previous research in females has been scant, though it is notable that one female only cohort did not see a rise in FGF21 following acute exercise [21] and another investigation was unable to study FGF21 in females due to baseline levels being below the limit of assay detection [22]. Slusher et al. included both men and women in their study of acute exercise and FGF21 and did not find any sex differences; however, this was not their main outcome variable and their sample size – one third of the present study – was likely too small to detect such differences [23]. One interesting study found that FGF21 levels increased post-exercise in pregnant women, but not in a similar non-pregnant cohort [24].

The lack of post-exercise FGF21 increase in females may offer a potential protective effect to reproductive and bone health. One study performed in mice showed that FGF21 played a role in disrupting female fertility [3]. While this finding has been called into question by a more recent article [25], FGF21 still appears capable of disrupting female fertility, even if it is not robust to all situations. In humans, FGF21 may be inversely related to bone density [26–28]. Since females are at a greater risk for osteoporosis than males, it is possible that a lack of FGF21 response to exercise may be an adaptive mechanism for maintaining bone mass. The precise mechanism by which FGF21 may be differentially regulated between females and males is a subject of future investigation. A hyper-release of estrogen does not appear to influence FGF21 [29] and the high levels of estrogen associated with pregnancy did not prevent pregnant women from experiencing increased FGF21 level post-exercise [24]; therefore, the difference is unlikely to be due to differences in estrogen. One of the primary biological differences between females and males is in muscle mass. While FGF21 may act as a myokine in some situations [30,31], circulating levels of FGF21 are believed to be of hepatic origin [32], particularly after exercise [7,9]. While liver mass differences are much closer between males and females [33] than skeletal muscle, a difference in liver size could at least be partially responsible. Future research could be directed towards finding mechanisms by which FGF21 may be differentially regulated between females and males.

The regulation of FGF21 is pluripotent and many upstream regulators of FGF21 are increased as a result of exercise. We investigated three biomarkers – glucagon, epinephrine, and glucose – to see if their changes were related to the FGF21 response. In contrast to prior studies [8,9] we did not see a significant elevation in glucagon post-exercise nor did we observe a significant correlation between IPE glucagon levels and FGF21. Although we did not see a significant correlation between glucagon IPE and the post-exercise FGF21 iAUC; both sexes did experience the highest average glucagon levels following the SS condition and this condition produced the highest FGF21 iAUCs for both sexes, which does not rule out the possibility that glucagon is still an important regulator of circulating FGF21 following exercise.

The hormone epinephrine is associated with FGF21 production [34]. A link between circulating epinephrine and circulating FGF21 in response to exercise has not been established. Consistent with prior investigations [10,21], we did not find a significant relationship between IPE epinephrine and post-exercise FGF21 iAUC.

The *FGF21* gene has a carbohydrate-response element-binding protein site [17], allowing for its transcription to be upregulated by carbohydrates. During the IE trial in males, blood glucose levels were significantly greater IPE than in the SS trial and strongly correlated with the FGF21 iAUC. The fact that IPE glucose was correlated to FGF21 in the IE condition, but not SS condition is an interesting finding. There might be a glucose threshold that must be exceeded in order for glucose to promote *FGF21* transcription. The fact that the post-exercise FGF21 iAUC was greater for the SS condition than IE condition, yet only correlated to blood glucose in the IE condition might indicate that FGF21 could be produced via multiple mechanisms during exercise or regulated differently by different types of exercise.

Circulating levels of FGF21 have been shown to differ in response to different types of exercise. As noted previously, FGF21 has been shown to consistently rise in response to steady state endurance exercise (at least in males), the same response has not been observed following resistance exercise [8,35]. Sprint interval exercise combines elements of both resistance and endurance exercise by alternating periods of high anaerobic energy demand with periods of low intensity recovery and has been shown to have similar metabolic benefits to steady state exercise [15,16]. Work by other researchers has shown variable findings when examining the blood FGF21 concentrations following sprint interval exercise [12,35]. However, we show that serum FGF21 concentrations are increased 1-hour after a bout of sprint interval exercise in healthy men. Previous research investigating the effect of steady state aerobic exercise in healthy men showed that exercising at a higher intensity was associated with a greater post-exercise FGF21 response [7,11,12]. We found a greater post-exercise iAUC in response to SS compared to IE, for both males and females; in males this difference was significant. This is in contrast to previous findings in young men [12]. Our results suggest that the post-exercise FGF21 response is not governed by exercise intensity alone as our participant’s experienced supra-aerobic intensities in the IE condition while experiencing a lesser FGF21 response in comparison to the SS condition. Thus, the metabolic pathways activated in aerobic exercise might more favorably promote FGF21 production.

Following exercise, the body continues to experience an elevated metabolic rate (EPOC). We found a similar magnitude of EPOC as measured by total kcals between the SS and IE conditions, despite the greater total volume of exercise performed in the SS condition. Intensity is generally believed to be more important than volume when it comes to EPOC magnitude [36,37] and interval exercise has been shown to produce a greater EPOC than steady state exercise when energetically balanced [38]. Therefore, it is interesting, but not surprising that we saw similar EPOC magnitudes between conditions.

In mice, exogenous administration of FGF21 is associated with increased caloric expenditure, increased fat oxidation and increased core temperature [39,40], all of which are also present during the EPOC response. Despite observing similar magnitudes of EPOC between the SS and IE conditions, we observed different post-exercise FGF21 iAUC values, indicating that FGF21 and EPOC are not linked. Furthermore, we did not observe any significant correlations between FGF21 and EPOC variables. We conclude that if FGF21 is related to the EPOC response, it is very minor. The lack of association between FGF21 and EPOC calls into question the metabolic benefits of the post-exercise rise in FGF21. Future investigations could be designed to determine the mechanism for this lack of association, by investigating factors such as fibroblast activating protein [41,42] or the FGF21 co-receptor β-klotho [39].

The present study is not without limitations. We used day counting as a method for determining menstrual cycle phase for female participants, which may not accurately reflect hormone levels [43]. FGF21 can be cleaved by the enzyme fibroblast activating protein, which is known to circulate in human plasma [41,42]. We did not investigate levels of fibroblast activating protein or distinguish between active and inactive forms of FGF21 which could alter FGF21 actions *in vivo*. We only monitored EPOC levels for one hour. While the majority of the excess energy expenditure resulting from EPOC occurs during the first hour, by not measuring EPOC until oxygen levels returned to baseline we missed some of the excess energy expenditure that may offer important insights. This study was conducted in a sample of 30 young, healthy individuals, which was significantly powered to detect differences between males and females, but not enough to detect differences in other genetic factors. Finally, FGF21 may behave differently in obese individuals [23], limiting the generalizability our findings.

## 5. Conclusion

In this study we present the novel finding that males experience a greater post-exercise FGF21 response than females. This finding may lead to a better understanding of the differences in metabolic health between sexes and it may suggest the need for different treatment pathways in the development of FGF21-analog drugs. We also show that continuous aerobic exercise is able to increase circulating levels of FGF21 to a greater extent than sprint interval exercise and that these changes are not associated with EPOC. Future research should further examine sex-based differences in FGF21 and explore the extent to which post-exercise increases in FGF21 influence metabolic health.

## Abbreviations

FGF21: Fibroblast Growth Factor 21
SS: steady state exercise
IE: sprint interval exercise
IPE: immediately post-exercise
iAUC: incremental area under the curve
tAUC: total area under the curve
BMI: body mass index
EPOC: excess post-exercise oxygen consumption.
